# Targeting TNF and Its Family Members in Autoimmune/Inflammatory Disease

**DOI:** 10.1155/2014/628748

**Published:** 2014-04-03

**Authors:** Sophie Desplat-Jégo, Linda Burkly, Chaim Putterman

**Affiliations:** ^1^Aix-Marseille Université, CNRS, NICN, UMR 7259, 13444 Marseille, France; ^2^AP-HM, Hôpital de la Conception, Service d'Immunologie, 13385 Marseille, France; ^3^Biogen Idec, Inc., 12 Cambridge Center, Cambridge, MA 02142, USA; ^4^Division of Rheumatology, Albert Einstein College of Medicine and Montefiore Medical Center, Bronx, NY 10461, USA

Tumor necrosis factor (TNF), a pleiotropic cytokine mainly produced by activated macrophages, modulates a wide range of biological functions in multiple tissues and organs. Besides its effects on tumor cell death, TNF is a key mediator of both acute and chronic inflammation. Since the description of TNF in the 1970s and through consideration of structural homologies, a total of 19 TNF-related cytokines have now been regrouped into a large family called the TNF ligand superfamily (TNFSF), whose members interact with TNF receptor superfamily (TNFRSF) members. More than 150,000 scientific publications (!) concerning TNF and its family members are available, demonstrating the strong interest of the scientific community in this molecule. Numerous studies implicating TNF family members in the pathophysiology of human autoimmune/inflammatory diseases have supported the emergence of TNF blocking agents developed for treatment of human disease, particularly over the last decade. These biotherapies, in the form of (I) chimeric, humanized, or human anti-TNF monoclonal antibodies or (II) fusion proteins involving a soluble TNF receptor, have been very successful in ameliorating disease signs and symptoms, especially in patients suffering from rheumatoid arthritis (RA) and Crohn's disease. Nonetheless, several aspects of these beneficial effects remain enigmatic. Moreover, the modulatory factors influencing TNF production by macrophages are not all known. Nevertheless, it is expected that over the next few years we will witness an increasing number of diseases for which TNF-blockade therapy is indicated.

In this special issue, eleven papers including research articles, review articles, and clinical studies provide new information and interesting discussion regarding current questions related to this hot topic.

In the first group of articles, interesting data is presented about TNF-blocking therapies and modulation of TNF generation by macrophages. Y. Lv et al. studied the nonneuronal cholinergic system existing in macrophages and show in a murine monocyte/macrophage cell line that bacterial lipopolysaccharide (LPS) exposure enhances autocrine acetylcholine production associated with an attenuation of TNF release. In this same cell line, K. Borzęcka et al. determined that, during high dose LPS stimulation, CD14 together with scavenger receptors is required for the binding of LPS but has a limited and dispensable contribution to TNF production. R. Cascão et al. recently identified gambogic acid as a simultaneous blocker of IL-1*β* and TNF secretion and described here a beneficial anti-inflammatory effect of gambogic acid in rat antigen-induced arthritis. Interestingly, F. R. Spinelli et al. show that blocking TNF biological effects during RA is associated with an increase of circulating endothelial progenitor cells, which might positively affect the endothelial function and may help in correcting the endothelial dysfunction observed in the disease.

In the second group of articles, possible off-label uses of anti-TNF therapy in various disorders are discussed. Due to their low prevalence, discussion of the use of anti-TNF modalities in Behçet's disease, sarcoidosis, and noninfectious uveitis in the review by D. Sánchez-Cano et al. was mainly based on case reports and case series. The clinical study of C. García-De-Vicuña et al. supports the usefulness of adalimumab in the treatment of refractory uveitis associated with juvenile idiopathic arthritis. P.-A. Jarrot and G. Kaplanski review the use of anti-TNF therapy in vasculitis and conclude that, except for Behçet's disease, this treatment has not shown significant efficacy. In their review article, A. Kumar et al. described how HIV infection is modulated by TNF and TNFR superfamily pathways and then discuss the emerging therapeutic options based on the modulation of TNF activity.

The third group of articles tackle the topic of the management of anti-TNF therapy for its well-established indications. B. Mörck et al. envision being able to reduce the drug costs in active HLA-B27 positive ankylosing spondylitis over time by reducing the infliximab dose and by extending the interval between infliximab doses. On the other hand, R. Altwegg and T. Vincent discuss the usefulness of monitoring serum trough levels and anti-drug antibodies in the optimization of anti-TNF therapies during inflammatory bowel disease.

Lastly, Y. Aiba and M. Nakamura open a debate concerning a putative therapeutic role of TL1A/DR3 inhibition. They suggest that the modulation of this particular TNF/TNFR superfamily member interaction may be a potential therapeutic target in several autoimmune diseases including inflammatory bowel disease, RA, ankylosing spondylitis, and primary biliary cirrhosis.

We hope that readers of the journal will find in this special issue not only accurate data and updated reviews on the targeting of TNF and its family members in autoimmune/inflammatory disease treatment, but also relevant questions that remain to be resolved including the extension of current therapeutic indications and the optimization of the anti-TNF therapies to find a better balance between cost and effectiveness.

*Sophie  Desplat-Jégo* *Sophie  Desplat-Jégo* *Linda  Burkly* *Linda  Burkly* *Chaim  Putterman* *Chaim  Putterman*

